# HIV-1, HSV-2 and syphilis among pregnant women in a rural area of Tanzania: Prevalence and risk factors

**DOI:** 10.1186/1471-2334-8-75

**Published:** 2008-06-02

**Authors:** Khadija I Yahya-Malima, Bjørg Evjen-Olsen, Mecky I Matee, Knut Fylkesnes, Lars Haarr

**Affiliations:** 1 Centre for International Health, University of Bergen, Bergen, Norway; 2 School of Nursing, Muhimbili University of Health and Allied Sciences, Dar es Salaam, Tanzania; 3 School of Public Health Nursing, Ministry of Health, Morogoro, Tanzania; 4 Haydom Lutheran Hospital, Mbulu District, Manyara, Tanzania; 5 Departments of Microbiology and Immunology, Muhimbili University of Health and Allied Sciences, Dar es Salaam, Tanzania; 6 The Gade Institute, Department of Microbiology and Immunology, University of Bergen, Bergen, Norway

## Abstract

**Background:**

Evidence suggests that a substantial proportion of new HIV infections in African countries are associated with herpes simplex virus type 2 (HSV-2). Thus, the magnitude of HSV-2 infection in an area may suggest the expected course of the HIV epidemic. We determined prevalence of genital herpes, syphilis and associated factors among pregnant women from a remote rural Tanzanian community that has a low but increasing HIV prevalence.

**Methods:**

We analysed 1296 sera and responses to a standard structured questionnaire collected from pregnant women aged between 15–49 years, attending six different antenatal clinics within rural Manyara and Singida regions in Tanzania. Linked anonymous testing (with informed consent) of the serum for specific antibodies against HSV-2 was done using a non-commercial peptide- 55 ELISA. Antibodies against syphilis were screened by using rapid plasma reagin (RPR) and reactive samples confirmed by *Treponema pallidum *haemagglutination assay (TPHA).

**Results:**

Previous analysis of the collected sera had shown the prevalence of HIV antibodies to be 2%. In the present study the prevalence of genital herpes and syphilis was 20.7% (95% CI: 18.53–23.00) and 1.6% (95% CI: 1.03–2.51), respectively. The presence of HSV-2 antibodies was associated with polygamy (OR 2.2, 95% CI: 1.62 – 3.01) and the use of contraceptives other than condoms (OR 1.7, 95% CI: 1.21 – 2.41). Syphilis was associated with reporting more than one lifetime sexual partner (OR 5.4, 95% CI: 1.88 – 15.76) and previous spontaneous abortion (OR 4.3, 95% CI: 1.52–12.02).

**Conclusion:**

The low prevalence of HIV infection offers a unique opportunity for strengthening HIV prevention in a cost-effective manner. The identification and control of other prevalent curable STIs other than syphilis and specific intervention of HSV-2 in specific populations like pregnant women would be one among approaches towards preventing incident HIV infections.

## Background

There is strong evidence that a substantial proportion of new HIV infections in African countries are associated with herpes simplex virus type 2 (HSV-2) [1-6]. Infection with HSV-2 has a significant impact on the risk of HIV acquisition and the impact increases as the HIV epidemic progresses [7,8]. One reason for this association could be the relative importance of HSV-2 in genital ulcer disease (GUD) in Africa has increased markedly. Infection with HSV-2 disrupts the genital mucosa and provides a portal of entry for HIV, leading to increased susceptibility of HIV in HIV-negative persons. In HIV-positive persons, infection with HSV-2 accelerates replication and genital shedding of the virus, thus such individuals are more likely to transmit HIV [9]. Studies from sub-Saharan countries have reported a high prevalence of HSV-2 in adults ranging from 30% to 80% in women and up to 50% in men [5,10,11]. In Tanzania, recent studies have shown HSV-2 represents 71% to 83% of all identified pathogens in genital ulcers [12,13]. Thus, the magnitude of HSV-2 infection may suggest the expected course of the HIV epidemic.

Tanzania is among the countries with a stabilised HIV epidemic. The estimated adult HIV prevalence was 8% by the end of 2004 based on data from the Tanzania HIV/AIDS indicator survey [14], closely matching HIV estimates (8.7%) reported from antenatal clinic surveillance in the same year [15]. The HIV prevalence (2%) in Manyara region by the end of 2004, matched with the estimate pregnant women attending local clinics within rural Manyara and Singida regions [14,16]. In the antenatal group, young women (15–19 years) presented with the highest prevalence (3.0%) and in the general population, men aged 35–49 years [16,17]. In comparison with other regions in Tanzania, the HIV prevalence is still low [14]. The magnitude of HSV-2 in the same area is largely unknown, while the prevalence of syphilis among pregnant women was 17% as reported by a previous study within the area [18]. Unlike genital herpes, studies exploring syphilis as a GUD enhancing HIV susceptibility and infectiousness in high-risk groups have revealed conflicting results [19,20]. The limitations have been due to either a few number of syphilis cases or case ascertainment by self-reports rather than laboratory confirmed results. In Tanzania, 51% of all stillbirths are associated with maternal syphilis [21]. Analysis of maternal syphilis and HIV prevalence among pregnant women attending selected antenatal clinics in by the end of 2004 showed geographical variation in the prevalence [22]. Rural antenatal clinics had high prevalence of syphilis (9.5%) and low HIV prevalence (5.2%) compared with other sites (6.8% syphilis vs. 9.3% for HIV). The high prevalence was associated with lack of formal education, inadequate access to health care and poverty [23,24]. Thus to determine the prevalence of syphilis and genital herpes in this rural area with low but increasing HIV prevalence, we analysed antibodies against HSV-2 and *Treponema pallidum*. We used data and serum samples collected from a previously reported antenatal survey [16].

## Methods

### Study area and population

The current study was part of the epidemiological study conducted within the remote rural areas of Manyara and Singida in Tanzania. The area falls within the service catchment area of Haydom Lutheran Hospital (HLH). The main focus aimed towards understanding the epidemiological context of HIV in the area as part of the comprehensive prevention-intervention programme launched by the hospital to combat HIV and other sexually transmitted infections in the area [25]. Part of the interventions included voluntary counselling and testing for HIV (VCT), syphilis and HSV-2. Education provided for participants included HIV- related preventive education and benefits of highly active antiretroviral treatment (HAART) for prevention of mother to child transmission (PMTCT) explained, according to national guidelines for care and treatment of people living with HIV and AIDS.

### Sampling

The survey included six antenatal clinics within the three administrative divisions that comprised the above-defined catchment area, between November 2003 and April 2004, and the study population is described in detail [16]. In brief, it included all counselled and consenting pregnant women aged between 15–49 years attending six local antenatal clinics (ANC) for the first time in that particular pregnancy during the surveillance period. Two clinics from each administrative division were included. Based on previous estimates of HIV, HSV-2 and syphilis prevalence in the area, HIV prevalence was the lowest, so the sample size was based on that estimate. A sample size of 1000 pregnant women would allow us to calculate a 2% prevalence of HIV with 95% confidence intervals of 1 to 3%. Point prevalence estimates of HIV, HSV-2 and syphilis are presented as the point estimate with their 95% confidence intervals.

### Data collection tools and laboratory methods

The research team collaborated with the hospital PMTCT teams in data collection. The PMTCT team was responsible for registration and coding of participant's identities and providing follow up care. The research team used a standard structured questionnaire to obtain information regarding socio-demographic and sexual behaviour characteristics. Included in the questionnaires were marital status, self-reported history of sexually transmitted infections and genital ulcers, the use of condoms and other contraceptives, and sexual partners. Blood was collected using vacutainers, and prepared serum samples were stored in the refrigerator and tested for HIV antibodies and syphilis the next day. Following HIV and syphilis antibody testing, the samples were stored at -80°C until analysis for HSV-2 antibodies. Screening for anti-HIV IgG antibody was performed anonymously using Bionor HIV-1 & 2 assay^® ^(BIONOR AS, Skien, Norway). Reactive samples were confirmed using Bionor HIV-1 & 2 assay^® ^(BIONOR AS, Skien, Norway). Screening for syphilis was done using Rapid plasma reagin (RPR) by Omega Immutrep-RPR^®^, Omega Diagnostics, Alva, UK and the reactive samples were confirmed by TPHA (Immutrep TPHA^®^, Omega Diagnostics, Alva, UK). Positive RPR and TPHA tests were considered to indicate current or untreated syphilis. Specific antibodies for HSV-2 were detected by the non-commercial peptide-55 Enzyme-linked immunosorbent assay as described elsewhere [26-28]. The assay is specific for reactivity with an epitop within glycoprotein G of HSV-2 (gG2) and does not cross-react with Herpes simplex virus type 1. Sensitivity and specificity of 100% and 98.4%, respectively, have been reported [27].

The cut-off value was set at a mean absorbance of the negative controls plus five standard deviations. Tanzanian sera, initially analysed by the same assay and the results confirmed with a commercial HerpeSelect 1 and 2 Immunoblot IgG ^® ^(IB0900G) from Focus Technologies Inc. (USA), were used as positive and negative controls [29]. The Regional Committee for Medical, Health Research, and Ethics in Western Norway (REK) and the Tanzanian National Institute of Medical Research (NIMR) gave ethical approval for research exploring HIV, HSV-2 and syphilis within the mentioned area. Regional and District level Ethical approval was sought and granted. Oral informed consent was sought from individuals and they where informed of their right to refuse to participate with out any consequences.

### Statistical analyses

Data were entered twice in Excel spreadsheets and analysed using Stata 9.2^® ^(College station, Texas, USA). We assessed self-reported previous adverse pregnancy outcomes as a probable indicator of previous syphilitic infection for women with previous pregnancies. The age at first pregnancy was explored as an indirect measure of unprotected sexual intercourse. The prevalence of HSV-2 and syphilis was compared to the previously reported HIV prevalence in the same population of pregnant women to assess the coexistence of these infections. We define polygamous relationship as women with sexual relations with a man having more than one wife or sexual partner(s) at the same time and monogamous relation women in marriage or sexual relationship with a man who has no other but only one wife/sexual partner at the same time.

Logistic regression was used to explore potential risk factors for HSV-2 and syphilis. Since HSV2 antibodies remain for life in infected people, older pregnant women would be more likely to have antibodies from previously acquired HSV2 infections. We adjusted for age in the analysis of HSV-2 and risk factors to avoid overestimation in older women and underestimation in younger women thus allowing comparison of the risk of HSV-2 infection.

Variables with p-values less than 0.25 on age-adjusted analysis were included in the final multivariate model and the associations presented as Odds ratios (OR) with a 95% confidence interval (CI).

## Results

In a sample of 1377 consenting pregnant women, 1296 women completed interviews and provided serum samples for laboratory testing. Four per cent of women (58/1377) declined a blood test, and we discarded 1.7% (23/1319) haemolysed samples. The mean age of the studied population was 26.3 (SD ± 6.1) years and the mean age at first pregnancy was 19 (SD ± 3) years. Ninety-one per cent of the women were married (Table 1) and 79% of them reported monogamous marriage while 28% of all women had no formal education.

**Table 1:** The prevalence of genital herpes and associated factors among pregnant women

**Characteristics**	**N (%)**	**HSV-2 prevalence % (95% CI)**	**Age-adjusted OR (95% CI)**	**Multivariate analysis OR (95% CI)**
**Total**	**1296**	**20.7 (18.53–23.00)**		
				
**Clinic**				
Basotu	242 (18.7)	7.0 (4.27 – 11.21)	1	1
Haydom	319 (24.6)	23.8 (19.33 – 28.86)	4.2 (2.41 – 7.31) *	4.2 (2.39 – 7.41) *
Muslur	192 (14.8)	32.8 (26.32 – 40.00)	6.5 (3.63 – 11.52) *	6.8 (3.76 – 12.27) *
Dangayda	113 (8.7)	32.9 (23.59 – 41.38)	6.0 (3.19 – 11.35) *	5.3 (2.78 – 10.16) *
Mwanga	249 (19.2)	20.1 (15.40 – 25.71)	3.3 (1.86 – 5.95) *	3.3 (1.84 – 6.02) *
Nduguti	181 (14.0)	14.4 (9.76 – 20.53)	2.2 (1.16 – 4.23) *	2.2 (1.15 – 4.24) *
				
**Age groups (years)**				
15–19	165 (12.7)	19.5 (13.91 – 26.58)	1	
20–24	417 (32.2)	19.4 (15.80 – 23.62)	1.0 (0.63 – 1.57)	0.8 (0.48 – 1.28)
25–29	349 (26.9)	17.4 (13.69 – 21.90)	0.9 (0.54 – 1.40)	0.7 (0.41 – 1.13)
30–34	224 (17.3)	24.5 (19.58 – 31.30)	1.3 (0.82 – 2.19)	0.9 (0.55 – 1.61)
≥35	141 (10.9)	27.7 (20.62 – 35.93)	1.6 (0.92 – 2.69)	1.0 (0.56 – 1.77)
				
**Marital status**				
Married	1183 (91.3)	20.9 (18.62 – 23.33)	1	__
Single/divorced/separated/widow	113(8.7)	18.6 (12.12 – 27.23)	1.1 (0.68 – 1.91)	
				
**Education (years)**				
None	349 (28.2)	20.9 (16.85 – 25.64)	1	__
1–6 years	174 (13.4)	17.8 (12.60 – 24.49)	0.8 (0.51 – 1.40)	
7 years or more	773 (59.7)	21.2 (18.42 – 24.30)	1.0 (0.74 – 1.9)	
				
**^a ^Polygamous relationship**				
No	1024 (79.0)	17.5 (15.23–19.90)	1	
Yes	272 (21.0)	32.7 (27.24–38.69)	2.2 (1.63 – 3.01) *	2.2 (1.62 – 2.99) *
				
**^b ^Age at first pregnancy**				
< 18 years	158 (14.0)	23.4 (17.21 – 30.94)	1	
≥ 18 years	974 (86.0)	20.4 (17.97 – 23.13)	1.2 (0.85–1.73)	1.2 (0.82 – 1.70)
				
**Number of lifetime sexual partners**				
One only	1231 (95.0)	18.2 (16.14 – 20.54)	1	
More than one	65 (5.0)	35.3 (24.36 – 47.90)	2.0 (1.21 – 3.46) *	**1.8 (1.04 – 3.04)**
				
**Use of contraceptives other than condom**				
No	1088 (84.0)	19.2 (16.93–21.71)	1	**1**
Yes	208 (16.0)	28.4 (22.45–35.09)	1.7 (1.20–2.37)*	1.7 (1.21–2.41) *
				
**History of sexually transmitted infections**				
No	1135 (87.6)	19.8 (17.56 – 22.29)	1	
Yes	161 (12.4)	27.0 (20.20 – 34.35)	**1.5 (1.01 – 2.17)**	1.3 (0.87–1.92)
				
**Ever used condom**				
No	1233 (95.1)	20.6(18.47 – 23.07)	1	__
Yes	63 (4.9)	20.6 (11.85 – 33.04)	1.1 (0.56 – 1.97)	

^a^Relationship with a man having more than one female sexual partner regardless of marital status

^b ^Analysed for those aged 20 ≥ years at the time of survey

Total HSV-2 positive (n = 268/1296)

*P-values: < 0.01

Table 1 summarizes results on HSV-2 prevalence and associated factors. The overall prevalence of HSV-2 was 20.7% (95% CI: 18.53–23.00), varying between 7.0% and 32.9%. HSV-2 prevalence increased with age, ranging from 19% in the 15–19 year age group to 27% among women aged 35 years or older. The prevalence was between 18% and 35% for all socio-demographic variables. Women in polygamous relationships, had high prevalence of HSV-2 (32.7%, 95% CI: 27.24–38.69) compared with women in monogamous relationships (17%, 95% CI: 15.23–19.90). HSV-2 prevalence did not differ with a history of condom use and only 4.9% of the women reported to have ever used condoms.

In age-adjusted analysis, the likelihood of HSV-2 antibodies was associated with a self-reported history of sexually transmitted infections, polygamous relationship, using non-condom contraceptives and more than one lifetime sexual partner. On multivariate analysis, all clinics except Basotu had high odds ratios of HSV-2 infection. Higher odds were particularly seen in Muslur (OR 6.8 (95% CI: 3.76–12.27) and Dangayda clinics (OR 5.3 (95% CI: 2.78 – 10.16), Table 1 right column. HSV-2 remained significantly associated with polygamous relationships, OR 2.2 (95% CI: 1.62 – 2.99) or the use of non-condom contraceptives OR 1.7 (95% CI: 1.21–2.41) and weakly associated with more than one lifetime sexual partner (OR 1.8 (95% CI: 1.04–3.04). We found no dual infections with HIV for those with HSV-2 antibodies (data not shown). Age at first pregnancy was not associated with increased risk of HSV-2 antibodies (Table 1).

The overall prevalence of syphilis was 1.6%, but was higher in women aged 20–24 years and 30–34 years (2.4% and 3.1%) (Table 2). The highest prevalence of syphilis (8.8%) was associated with more than one lifetime sexual partner, followed by self-reported spontaneous abortion 6.1%. Women in polygamous relationships and those with a self-reported history of sexually transmitted infection had a prevalence of 3.7% (95% CI: 1.88–6.87) and 3.1% (95% CI: 1.15–7.48), respectively. None of those who had ever used condoms had antibodies against syphilis. Prevalence of syphilis in women aged less than 18 at first pregnancy was 2.5% (Table 2) and 1.7% for pregnancies at older age.

**Table 2:** The prevalence of syphilis and associated factors among pregnant women

**Characteristics**	**N (%)**	**Syphilis prevalence % (95% CI)**	**Age-adjusted OR (95% CI) (p-value)**	**Multivariate analysis OR (95% CI) (p-value)**
**Total**	**1296**	**1.6 (1.03 – 2.51)**		
				
**Age groups (years)**				
15–19	165 (12.7)	0	__	__
20–24	417 (32.2)	2.4 (1.22 – 4.51)		
25–29	349 (26.9)	0.9 (0.22 – 2.69)		
30–34	224 (17.3)	3.1 (1.38 – 6.60)		
≥35	141 (10.9)	0.7 (0.03 – 4.47)		
				
**Marital status**				
Married	1183 (91.3)	1.8 (1.13 – 2.75)		
Single/divorced/separated/widow	113 (8.7)	0	__	__
				
**Education (years)**				
None	349 (28.2)	2.3 (1.07 – 4.64)	1	
1–6 years	174 (13.4)	1.7 (0.45 – 5.36)	0.8 (0.20 – 2.85)	
7 years or more	773 (59.7)	1.3 (0.66 – 2.48)	0.6 (0.22 – 1.43)	__
				
**Polygamous relationship**				
No	1024 (79.0)	1.1 (0.57 – 1.98)	1	**1**
Yes	272 (21.0)	3.7 (1.88 – 6.87)	**2.7 (1.08 – 6.64) (p = 0.03)**	**2.6 (1.06 – 6.51) (p = 0.03)**
				
**^a ^Age at first pregnancy**				
< 18 years	158 (14.0)	2.5 (0.81 – 6.76)	__	
≥ 18 years	974 (86.0)	1.7 (1.05 – 2.84)		
				
**Outcome of previous pregnancy (n = 1155)**				
Live child	916 (79.3)	1.3 (0.71 – 2.34)	1	1
Stillbirth	140 (12.1)	2.1 (0.55 – 6.62)	1.6 (0.45 – 5.92)	2.4 (0.64 – 8.95)
Spontaneous abortion	99 (8.57)	6.1 (2.49 – 13.24)	**4.9 (1.78 – 13.25) (p = 0.00)**	**4.3 (1.52 – 12.02) (p = 0.00)**
				
**Number of lifetime sexual partners**				
One only	1231 (95.0)	1.2 (0.71 – 2.05)	1	**1**
More than one	65 (5.0)	8.8 (3.64 – 18.85)	**7.8 (2.94 – 20.86) (p = 0.00)**	**5.4 (1.88 – 15.76) (p = 0.00)**
				
**Use of contraceptives other than condom**				
No	1088 (84.0)	1.8 (1.16 – 2.88)	1	1
Yes	208 (16.0)	0.5 (0.02 – 3.06)	0.3(0.03 – 1.93)	0.3 (0.04 – 2.66)
				
**History of sexually transmitted infections**				
No	1135 (87.6)	1.4 (0.84 – 2.33)	1	1
Yes	161 (12.4)	3.1 (1.15 – 7.48)	2.2 (0.81 – 6.20)	1.8 (0.60 – 5.18)
				
**Ever used condom**				
No	1233 (95.1)	1.7 (1.08 – 2.64)	__	__
Yes	63 (4.9)	0		

Total syphilis positive (n = 21/1296). Results for analysis of syphilis prevalence by clinic locations are not shown due to very small numbers or none-to give any substantial statistical inference.

^a ^Analysed for those aged 20 ≥ years at the time of survey (n = 1132)

In multivariate analysis, women who reported previous spontaneous abortion as an adverse pregnancy outcome, were more likely to have a positive syphilis serology (OR 4.3, 95% CI: 1.52–12.02) compared with those reporting stillbirth (OR 2.4, 95% CI: 0.64–8.95). Odds were also higher for women who had more than one lifetime sexual partner, OR 5.4 (95% CI: 1.88–15.76). Dual syphilis and HIV infection occurred in only one individual aged 30 years and this person had had more than one lifetime sexual partner. Eight of the 21 women with syphilis were infected with HSV-2 (OR 2.5, 95% CI: 0.32 – 19.36), but the figures are too small to interpret the precise association. In addition, being less than 18 years at first pregnancy did not have a significantly increased risk of positive syphilis serology.

## Discussion

The overall prevalence of HSV-2 antibodies among pregnant women in the rural Manyara and Singida regions in Tanzania was 20.7%, varying from 7% to 33% between clinics and increasing with age. This is a slightly lower prevalence than observed by Obasi and colleagues in a different rural region in Tanzania (Mwanza) where 42% of the women had HSV-2 antibodies [30]. The prevalence of syphilis in the present study (1.6%) is much lower than previously observed (17%) in this region, the finding may partly reflect the effect of syndromic management of STI imposed in health services since 1999 [31]. Our findings though suggestive of the relatively low HSV-2 and syphilis prevalence in the area compared to other places in Tanzania, point out a potential opportunity for indirect HIV prevention by controlling prevalent STIs.

Measurement of the HSV-2 antibodies was performed with a non-commercial method using a branched peptide with a sequence similar to a specific epitope in glycoprotein G of HSV-2 [26,28]. Commercial assays use the entire glycoprotein G, and may sometimes have problems with false positive results. When comparing performance of peptide 55 method with Western blot, we did not detect any false positive results in Tanzanian sera from persons 13 years old or older [32].

Studies from rural areas in Gambia in West Africa, using the peptide 55 method, have shown infection in 30% of the studied women [33,34]. Other prevalence studies in urban regions of Tanzania have shown that 35% of pregnant women in Dar es Salaam [35] and 39% of women attending primary health care clinics in Moshi were infected with HSV-2 [4]. Among STD patients in urban Mbeya and Dar es Salaam regions of Tanzania, HSV-2 was the most commonly (71%) identified agent in genital ulcer specimens [12]. In these patients, detection of HSV-2 was at significantly higher in HIV infected compared to those not infected [12]. Therefore, taken together, these results indicate a generalised HSV-2 infection in the Tanzanian population. Suggestions on the limited impact of HSV-2 on incident HIV infections in the early stages of the HIV epidemic may also hold true for this area [7]. Despite the generalised, stable HIV epidemic in Tanzania, the epidemiological patterns of STIs have shown regional variations on the level and magnitude, largely influenced by cultural, economical political and social factors. [14,17]. The prevalence of HSV-2 was significantly higher in all clinics compared to Basotu clinic but this village reportedly, had the highest HIV prevalence [17]. We are uncertain to the reasons that might explain this distribution. Basotu presents a very mixed population compared to the other villages, Lake Basotu may attract fishermen from other regions and in-migration of HIV infected persons is possible. Fishing provides a substantial economical gain that is lacking in other villages.

Our definition of positive syphilis serology would not only include those with infectious syphilis (primary and secondary), but also cases of untreated latent syphilis though persons in the latent syphilitic state are non-infectious. Reported estimates of the prevalence of syphilis in other rural antenatal sentinel sites in Tanzania in 2004 ranged from 14.9% in Kagera and 2.1% in Kigoma region. The corresponding prevalence of HIV was 4.7% and 5.1% respectively [15]. The later study performed RPR under field conditions, and lacked confirmatory tests for syphilis and likely to have overestimated prevalence of syphilis. Likewise, Kagera region is experiencing a different stage of the HIV epidemic compared to rural Manyara, Singida and Kigoma regions. Kagera was the first region in Tanzania to report AIDS cases and experienced a severe HIV epidemic (24.2%) since 1987 and is low levelling off as a result of excessive mortality and multiple interventions [36]. While rural Manyara, Singida and Kigoma regions had relatively lower HIV prevalence in the last decade but have recently shown an increasing potential. Thus, the interpreting compared prevalence of syphilis with rural Manyara and Singida region is with caution. The proportion of active syphilis in our study might be lower than our prevalence estimates by using TPHA, since antibodies detected by this method remain for life. While, the use of Venereal Disease Research Laboratory (VDRL) test alone in the previous local study in 1999 might have overestimated the prevalence [18].

The low prevalence of HIV in our study (2.0%) may explain why we did not detect an association between infections with HIV and HSV-2. The potential reason for the low prevalence was associated with remoteness and reduced mobility of the people within this rural area. High numbers of lifetime sexual partners have previously shown to increase the risk of acquiring infection with HSV-2 and syphilis [4,35,37]. These risk factors are also presented in this study, as shown in Tables 1 and 2. Women in polygamous relationships presented with higher prevalence of HSV-2 (Table 2), in agreement with a report from Gambia [11]. In the present work as well as in others syphilis was shown to be a high probable risk for spontaneous abortion (Table 2) [38,39]. However, we did not find an association with stillbirth, even though 25% of the pregnancies might result in stillbirth in cases of maternal syphilis [21]. The lack of association in our study could be, partly due to confounding effects of other causes of stillbirth, beyond the scope of this study and the lower number of syphilis cases. Although sexual transmission of syphilis is usually limited to the early stages, vertical transmission is still possible in latency.

## Conclusion

Though we cannot rule out the effect of other STIs on the level HIV infection presented, the identified risk factors for STI are similar to other areas in Tanzania that have experienced a higher level of HIV and related STIs. The low but changing prevalence of HIV infection offers a unique opportunity for strengthening HIV prevention in a cost-effective manner [40]. The identification and control of other prevalent curable STIs other than syphilis and specific intervention for HSV-2 in specific populations like pregnant women should be among approaches towards preventing incident HIV infections.

## Competing interests

The authors declare that they have no competing interests.

## Authors' contributions

All authors contributed substantially to the paper. All authors conceived the study. The first author conducted the study and performed HSV-2 serology, supervised by LH. BE-O and MIM supervised the fieldwork. KIY–M did the statistical analysis and interpretation. All authors helped to conceptualise ideas, interpret the findings and reviewed drafts of the manuscript.

## Pre-publication history

The pre-publication history for this paper can be accessed here:


